# Structural Dynamics of the Ubiquitin Specific Protease USP30 in Complex with a Cyanopyrrolidine-Containing Covalent Inhibitor

**DOI:** 10.1021/acs.jproteome.4c00618

**Published:** 2025-01-13

**Authors:** Darragh P. O’Brien, Hannah B.L. Jones, Yuqi Shi, Franziska Guenther, Iolanda Vendrell, Rosa Viner, Paul E. Brennan, Emma Mead, Tryfon Zarganes-Tzitzikas, John B. Davis, Adán Pinto-Fernández, Katherine S. England, Emma J. Murphy, Andrew P. Turnbull, Benedikt M. Kessler

**Affiliations:** † Target Discovery Institute, Centre for Medicines Discovery, Nuffield Department of Medicine, University of Oxford, Oxford, OX3 7FZ, U.K.; ‡ Thermo Fisher Scientific, San Jose, California, California 95134, United States; § ARUK-Oxford Drug Discovery Institute, Centre for Medicines Discovery, Nuffield Department of Medicine, University of Oxford, Oxford OX3 7FZ, U.K.; ∥ Chinese Academy of Medical Sciences Oxford Institute, Nuffield Department of Medicine, University of Oxford, Oxford OX3 7BN, U.K.; ⊥ Cancer Research Horizons, Francis Crick Institute, London NW1 1AT, U.K.

**Keywords:** mitophagy, ubiquitin specific protease USP30, cyanopyrrolidine inhibitors, activity-based protein profiling mass spectrometry, enzyme kinetics, Hydrogen−Deuterium eXchange-Mass spectrometry, molecular docking

## Abstract

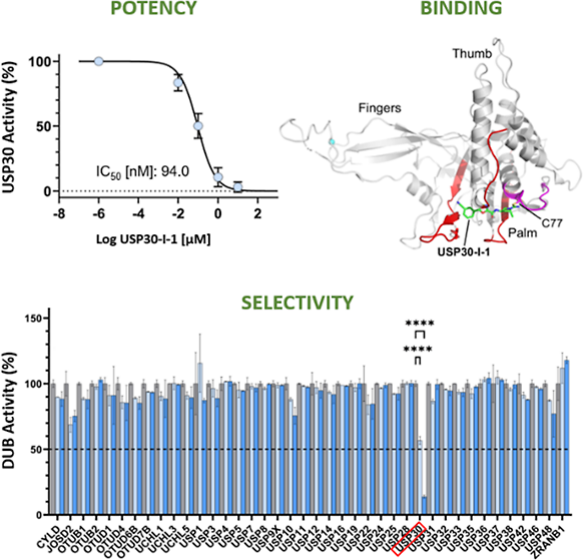

Inhibition of the mitochondrial deubiquitinating (DUB) enzyme USP30 is neuroprotective and presents therapeutic opportunities for the treatment of idiopathic Parkinson’s disease and mitophagy-related disorders. We integrated structural and quantitative proteomics with biochemical assays to decipher the mode of action of covalent USP30 inhibition by a small-molecule containing a cyanopyrrolidine reactive group, **USP30-I-1**. The inhibitor demonstrated high potency and selectivity for endogenous USP30 in neuroblastoma cells. Enzyme kinetics and hydrogen–deuterium eXchange mass spectrometry indicated that the inhibitor binds tightly to regions surrounding the USP30 catalytic cysteine and positions itself to form a binding pocket along the thumb and palm domains of the protein, thereby interfering its interaction with ubiquitin substrates. A comparison to a noncovalent USP30 inhibitor containing a benzosulfonamide scaffold revealed a slightly different binding mode closer to the active site Cys77, which may provide the molecular basis for improved selectivity toward USP30 against other members of the DUB enzyme family. Our results highlight advantages in developing covalent inhibitors, such as **USP30-I-1**, for targeting USP30 as treatment of disorders with impaired mitophagy.

## 1 Introduction

Ineffective repair or clearance of damaged mitochondria negatively affects cellular health and is an integral feature of several neurodegenerative disorders, such as Parkinson’s Disease (PD), Alzheimer’s Disease, and amyotrophic lateral sclerosis, in addition to cardiomyopathy and aging.^1−3^ Without their elimination, defective mitochondria accumulate, resulting in the excessive production of highly reactive and toxic oxygen species.^4,5^ This results in extensive damage to the overall cellular integrity and survival. Mitophagy, the cell’s specialized quality control system for the clearance of damaged mitochondria, can proceed through the concerted action of two enzymes; the mitochondrial outer membrane (MOM)-associated ubiquitin (Ub) serine/threonine kinase PINK1 and the cytoplasmic E3 ligase Parkin.^6^ PINK1 and Parkin activity leads to the hyperubiquitination of damaged proteins on the MOM, providing the molecular signal for their removal. Mitophagy is negatively regulated by several deubiquitinating (DUB) enzymes, including ubiquitin specific proteases (USP) 8, 14, 15, 30, and 35.^7−11^ The central action of these DUB enzymes is to remove Ub moieties from mitochondrial proteins and, as a consequence, modulate mitophagy. Of these, USP30 has been associated with mitophagy, likely due to its exclusive expression on the MOM.^12^ Aside from mitochondria, its analogous expression on peroxisomes widely implicates it in pexophagy and redox homeostasis.^13,14^

An accumulation of damaged mitochondria has been linked to both familial and sporadic forms of PD. Loss-of-function mutations in PINK1 and PRKN genes lead to a buildup of defective mitochondria and a gradual loss of dopaminergic neurons in the basal ganglia, resulting in a rare and inherited form of early onset Parkinsonism.^15,16^ Inhibiting USP30 may therefore boost a mitochondrial turnover in both early onset Parkinsonism and in PD, at least in this population of patients, offering a new strategy for the treatment of the disease and similar neurodegenerative conditions. This has justifiably attracted a great deal of attention in both academia and the industry, with several small-molecule inhibitors which can chemically reduce USP30 activity in development.^17−22^ Significantly, the USP30 inhibitors MTX652 and MTX325 are currently being tested in clinical trials for the treatment of acute kidney injury and PD.^23^

We have recently described the structural interplay of USP30 following complex formation with a noncovalent benzosulfonamide inhibitor “**USP30**_**inh**_”, which has been shown to boost mitophagy in dopaminergic neurons by reducing USP30 activity.^19,22,24^ A combination of activity-based protein profiling mass spectrometry (ABPP-MS), biolayer interferometry, hydrogen/deuterium eXchange mass spectrometry (HDX-MS), and computational modeling allowed us to comprehensively profile the potency, selectivity, and mechanism of inhibition of **USP30**_**inh**_ for dampening endogenous USP30. This was the first study of its kind to provide detailed structural and mechanistic information on the interaction of USP30 with any active small-molecule drug targeting it.

We now extend our study to structurally profile USP30s interaction with a small, covalent, cyanopyrrolidine scaffold-containing inhibitor, **USP30-I-1** (patent WO2020212350A1; Mission Therapeutics).^25,26^ Covalent inhibitors possess significant advantages over their noncovalent counterparts, including a more prolonged duration of action and the formation of more specific and irreversible bonds with their substrates, enhancing the overall drug efficacy.^27^ Newer-generation USP30 inhibitors are therefore likely to harness covalent bond formation between the compound and protein. Using a biophysical and structural proteomics approach similar to our previous work,^24^ we show that **USP30-I-1** is highly selective and potent against endogenous USP30 when compared to the >40 other endogenous DUBs identified in the neuroblastoma-derived SH-SY5Y cell line. Covalent **USP30-I-1** binds to USP30 with a similar affinity to the previously characterized noncovalent **USP30**_**inh**_, with binding primarily restricted to a small region covering the USP30 active site cysteine residue. Several regions along the palm and thumb domains of the protein also become solvent protected in the presence of **USP30-I-1**. Directly comparing the two studies, we decipher commonalities and differences between covalent and noncovalent mechanisms of USP30 inhibition. Our biochemical, structural, computational, and biophysical data empower the design of next-generation USP30 inhibitors to further drive drug discovery campaigns in the USP30 inhibitor space.

## 2 Experimental Section

### 2.1 USP30 Inhibitor Purity

**USP30-I-1** has a cyanopyrrolidine warhead for the covalent reaction with Cys77 of USP30. The compound was 93% pure at 254 nm UV and 100% by ELSD (Figure S1). The observed MW/[M + H]^+^ 321.30 and Theoretical 321.30. USP30 inhibitor **USP30-I-1** synthesis and characterization was reported previously (patent WO2020212350A1; Mission Therapeutics).^23,25,26^ The inhibitor was confirmed to bind to recombinant USP30 by RapidFire MS analysis (Figure S2).

### 2.2 ABPP Assay

#### 2.2.1 Cell Culture and Lysis

SH-SY5Y cells were cultured and lysed as previously reported.^24^ Briefly, cells were cultured at 37 °C in 5% CO_2_ in a Eagle’s minimum essential medium and Ham’s F12 nutrient mix supplemented with fetal bovine serum, nonessential amino acids, and Glutamax. Cells were washed with phosphate-buffered saline, scraped, and collected by centrifugation at 200*g*. Pellets were resuspended in 50 mM Tris base, 5 mM MgCl_2_·6H_2_O, 0.5 mM EDTA, 250 mM sucrose, and 1 mM DTT (pH 7.5) and lysed with glass bead beating. After 4 °C clarification at 600*g* for 10 min, lysate protein concentrations were measured by a bicinchoninic acid assay.

#### 2.2.2 HA-Ub-PA Activity-Based Probe Profiling

HA-Ub-PA was synthesized as outlined previously.^28,29^ Methodology for HA-Ub-PA activity-based probe profiling was described in our previous study on a USP30 noncovalent inhibitor.^24^ Briefly, SH-SY5Y lysates were incubated for 1 h at 37 °C with either **USP30-I-1** or dimethyl sulfoxide (DMSO) at the indicated concentrations in duplicate. Labeling with HA-Ub-PA was then carried out at 37 °C for 45 min. The reaction was then quenched with SDS and NP-40 and diluted in 50 mM Tris, 0.5% NP-40, 150 mM NaCl, and 20 mM MgCl_2_·6H_2_O, pH 7.4. HA-Ub-PA-bound DUBs were immunoprecipitated overnight at 4 °C with end-overend rotation using 150 μL of anti-HA agarose slurry. HA-Ub-PA DUB complexes were eluted with 2x Laemmli buffer, reduced, alkylated, and cleaned up for LC–MS/MS using S-Trap micro columns.^30^ Samples were digested with 2 μg of trypsin. Eluates were dried and resuspended in 0.1% formic acid (FA).

#### 2.2.3 Western Blot Quantitation

Densitometry image analysis was applied for Western blot quantification of the unbound and HA-Ub-PA-bound USP30 bands. The percentage of signal from the HA-Ub-PA-bound USP30 band relative to signal from both bands was used to quantify USP30 activity and inhibition. Activity was normalized relative to that of the negative (no HA-Ub-PA) and positive (HA-Ub-PA) controls. The IC_50_ was extracted from fitting **USP30-I-1** inhibition quantitation with the equation: *Y* = 100/(1 + 10 × (*X* – Log IC_50_)) in Prism (version 10.1.1).

#### 2.2.4 LC–MS/MS Data Collection

Samples were analyzed by LC–MS/MS using the Vanquish Neo UHPLC (Thermo) connected to a Thermo Orbitrap Ascend mass spectrometer (Thermo). The Vanquish Neo was operated in “Trap and Elute” mode using a PepMap Neo trap (185 μm, 300 μm × 5 mm) and an EASY-Spray PepMap Neo column (50 cm × 75 μm, 1500 bar). Tryptic peptides were separated over a 60 min linear gradient of 3 to 20% B—80% ACN, 0.1% FA in 40 min, and 20 to 35% B in 20 min. The system was maintained at a flow rate of 300 nL/min flow rate. Samples were analyzed by tandem mass spectrometry as previously described.^24,31,32^ Data were collected on an Orbitrap Ascend Tribrid mass spectrometer (Thermo Scientific) over *m*/*z* 350–1650 by Data Independent Acquisition (DIA), with a 50 K resolution, a maximum injection time of 91 ms, an AGC set to 125%, and a radio frequency lens set to 30%. MS2 data were collected using the tMSn scan function, with 40 DIA scan windows of variable widths, an Orbitrap resolution of 30 K, a normalized AGC target of 1000%, a maximum injection time set to auto, and a collision energy set to 30%.

#### 2.2.5 LC–MS/MS Data Processing

LC–MS/MS data was processed using DIA-NN (version 1.8.1) for a library free search using a *Homo sapiens* Uniprot database (20,416 entries, retrieved February 15th, 2023) with all settings left as default.^33^ The unique gene matrix output was used to identify DUBs that were HA-Ub-PA enriched, with intensities 5-fold higher in the HA-Ub-PA positive control when compared to the negative control with no HA-Ub-PA. YOD1 and USP9Y were removed from analysis, as they were not consistently identified across replicates. Significant inhibition of DUBs was identified using two-way ANOVA analysis in Prism (version 10.1.1) with the Dunnett method for multiple correction testing (****p < 0.0001).

### 2.3 Enzyme Kinetics

#### 2.3.1 USP30 Activity Assay

Fluorescence intensity measurements were used to monitor the cleavage of a ubiquitin-rhodamine substrate. All activity assays were performed in black 384-well plates in assay buffer (20 mM Tris–HCl, pH 8.0, 150 mM Potassium Glutamate, 0.1 mM TCEP and 0.03% Bovine Gamma Globulin) with a final assay volume of 20 μL. A concentration of 0.2 nM USP30 [residues 64-502Δ179-216 and 288-305, Viva Biotech (Shanghai) Ltd.] was added and preincubated with **USP30-I-1** for 30 min. A total of 25 nM ubiquitin-rhodamine 110 (Ubiquigent) was added to initiate the reaction and the fluorescence intensity was recorded for 30 min on a PherastarFSX (BMG Labtech) with an Ex485/Em520 optic module. Initial rates were plotted against compound concentration to determine the IC_50_.

#### 2.3.2 Kinetic Assays—Determination of *k*_inact_/*K*_*i*_

Kinetic assays were performed in a 384-well Sensoplate in assay buffer with a final assay volume of 50 μL. The assay was carried out using 5 nM USP30 and reactions were started by simultaneous addition of 180 nM ubiquitin-rhodamine 110 to all 384 wells using the FLIPR Tetra (Molecular Devices) dispense function. Fluorescence was monitored every 3 s (excitation wavelength 470–495 nm, emission 515–575 nm, camera gain 70, exposure time 0.6 s, and excitation intensity 80%) over 10 min.

Analysis was performed in GraphPad Prism. Fluorescence intensity was plotted vs inhibitor concentration at each time point to determine IC_50_, IC_50_ was then plotted vs incubation time and fitted to eq 1 (Krippendorf)^34^ to obtain the inhibition constant *K*_I_ and the rate of enzyme inactivation *k*_inact_, the values can be used to obtain *k*_inact_/*K*_I_, a second-order rate constant describing the efficiency of covalent bond formation.
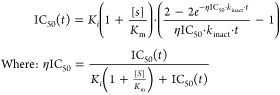
1

*K*_inact_/*K*_I_ was also determined using the traditional method of fitting the progress curves to eq 2 and plotting *k*_obs_ vs [**USP30-I-1**] and fitting with eq 3 (supplemental).
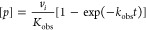
2
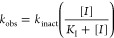
3

### 2.4 HDX-MS

#### 2.4.1 Materials

The same recombinant USP30 construct (residues 64-502Δ179-216 and 288-305, Viva Biotech (Shanghai) Ltd.) that was used in our in vitro enzyme kinetics analysis was also used for HDX-MS. LC–MS grade water, LC–MS grade 0.1% FA, and LC–MS grade ACN with 0.1% FA in water were purchased from Fisher Scientific (Hampton, NH). Guanidine hydrochloride 8 M solution, TCEP-HCl, and DMSO were purchased from Thermo Scientific (Rockford, IL). Citric acid, sodium chloride, and HEPES were purchased from Sigma-Aldrich (St. Louis, MO). Deuterium oxide (99+ %D) was purchased from Cambridge Isotope Laboratories (Tewksbury, MA).

#### 2.4.2 Sample Preparation

Initial stock concentrations of USP30 and compound **USP30-I-1** in DMSO were 66 μM and 10 mM, respectively. For in-solution HDX-MS, a working sample of USP30 and **USP30-I-1** (i.e., the holo-USP30) was prepared by a volume-to-volume mixture at a molar ratio of 1:2 (USP30:**USP30-I-1**) and diluted to a nominal concentration of 11 μM for the USP30 and 22 μM for **USP30-I-1**. The reference state (i.e., apo-USP30) was 11 μM USP30 protein, which was supplemented with DMSO in place of the compound.

#### 2.4.3 HDX LC–MS/MS Data Collection

In-solution HDX-MS was performed as follows: the labeling buffer was 50 mM HEPES pD 7.2, 400 mM NaCl, and 2 mM TCEP in D_2_O, and the quench buffer was 2 M guanidine HCl and 100 mM citric acid, pH 2.3, in water. The pH of the labeling buffer was measured and corrected to pD (pD = pH + 0.4). Approximately, 3.5 μL of the USP30 with **USP30-I-1** complex mixture was diluted with the labeling buffer (1:20 ratio, achieving an excess D_2_O concentration of 95%) and incubated in D_2_O buffer at 20 °C for 30, 60, 600, and 3600 s in triplicate. Nondeuterated controls were prepared in an identical manner, except H_2_O was used in place of D_2_O in the labeling step. Then, the labeled sample was quenched by adding quench buffer (1:1 ratio) to achieve a final pH of 2.5. LC/MS bottom-up HDX was performed using a Thermo Scientific Ultimate 3000 UHPLC system and a Thermo Scientific Orbitrap Exploris 480 Hybrid mass spectrometer. The quenched samples (50 μL) were digested with a pepsin/proteaseXIII (NovaBioAssays, MA) column (2.1 × 3.0 mm) at 8 °C for 3 min and trapped in a 1.0 mm × 5.0 mm, 5.0 μm trap cartridge (Thermo Scientific Acclaim PepMap100) for desalting. Peptides were separated on a Thermo Scientific Hypersil Gold, 50 × 1 mm, 1.9 μm, C18 column with a linear gradient of 10% to 40% Buffer B (A: water, 0.1% FA; B: ACN, 0.1% FA) at a flow rate of 40 μL/min. Pepsin wash was performed for each run to limit carry-over. To limit back-exchange, the quenching, trapping, and separation steps were performed at near 0 °C. Labeling, quenching, and online digestion were performed in a fully automated manner with a Chronnect HDX workstation by Trajan.

#### 2.4.4 Data Analysis

Before conducting the HDX-MS experiment, an unspecific digested peptide database was created for a nondeuterated USP30 sample using data-dependent and targeted HCD-MS^2^ acquisition. Peptide identification was performed using BioPharma Finder (v5.1). HDX-MS data files were processed and manually curated with the USP30 peptide database using HDExaminer by Trajan. A single charge state with high quality spectra for all replicates across all HDX-MS labeling times was chosen to represent the HDX for each peptide. A hybrid statistical significance approach was performed afterward using an in-house MATLAB script.^35^ The significant differences observed at each residue was used to map HDX-MS consensus effects (based on overlapping peptides) onto the catalytic domain using the X-ray structure of human USP30 in complex with a Fab fragment antibody and covalent inhibitor, 552, as the template (PDB code: 8D1T; unpublished).

### 2.5 Molecular Docking

We performed molecular docking simulations using the covalent docking method in ICM-Pro (MolSoft LLC). There are two inhibitor-bound X-ray structures for human USP30 in the Protein Data Bank, corresponding to the catalytic domain in complex with a Fab fragment antibody and covalent inhibitors 552 (PDB code: 8D1T; unpublished) and 829 (PDB code: 8D0A; unpublished), at 2.94 and 3.19 Å resolution, respectively. The highest resolution human USP30 catalytic domain inhibitor complex structure (corresponding to PDB code: 8D1T) in which the Fab fragment antibody and covalent inhibitor, 552, had been removed, was used as the target receptor for docking studies with **USP30-I-1**. The in silico binding pose of **USP30-I-1** with the best docking score is shown in Figure 3.

## 3 Results and Discussion

### 3.1 **USP30-I-1** Targets Endogenous USP30 in a Highly Potent and Selective Manner

**Figure 1:**
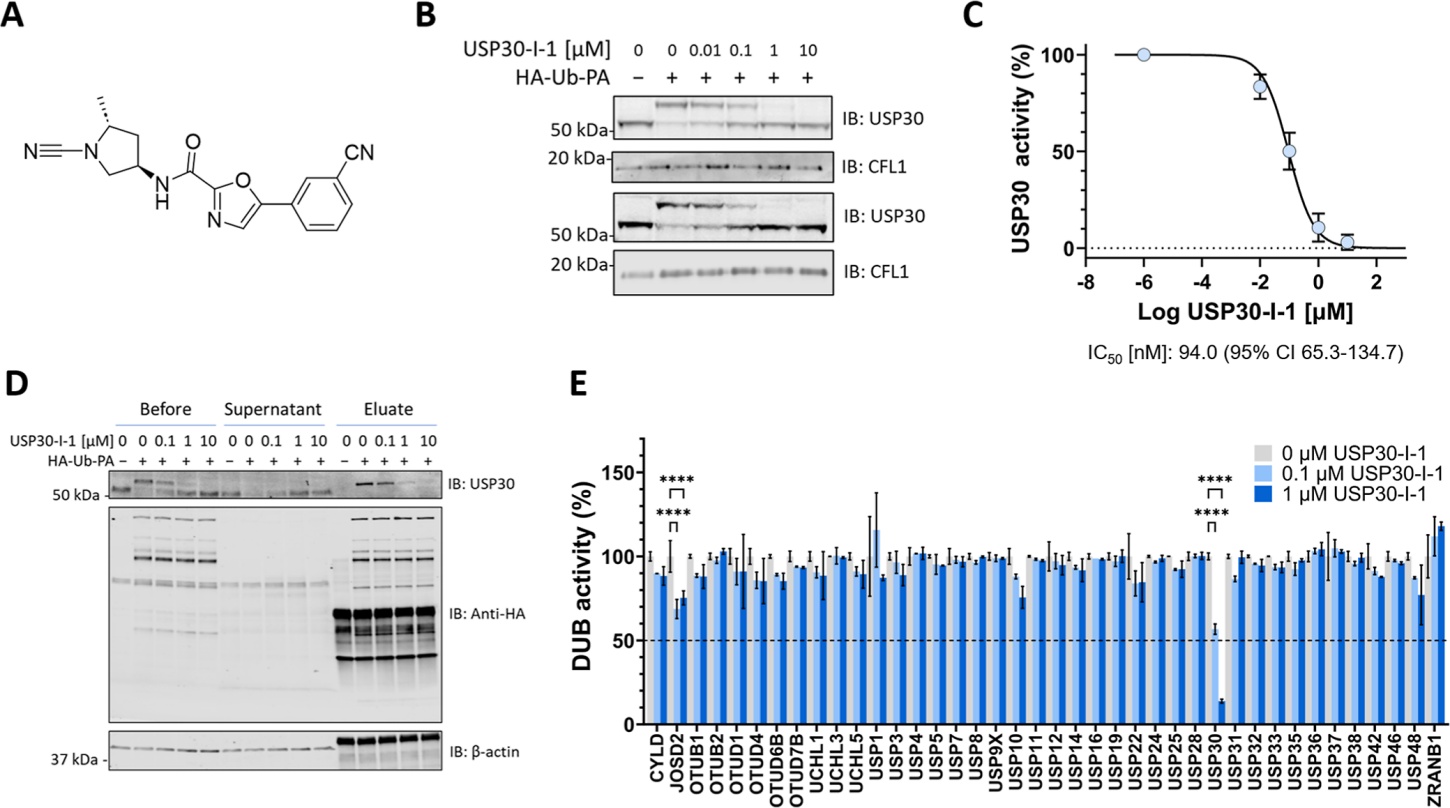
**USP30-I-1** is selective and potent for endogenous USP30. (A) Structure of **USP30-I-1**. (B) *n* = 2 Western blot of USP30 with ∼10 kDa mass increase showing HA-Ub-PA bound to USP30 and prevention of HA-Ub-PA binding with increasing **USP30-I-1** concentrations. (C) Densitometric quantitation of USP30 HA-Ub-PA labeling in B, fit to *Y* = 100/(1 + 10 × ((*X* – Log IC_50_))) for IC_50_ value extraction. (D) HA-Ub-PA binding to USP30 and prevention by **USP30-I-1** as shown in B. The supernatant and eluate of a HA immunoprecipitation shows efficient pull down of USP30 with HA-Ub-PA labeling, and a reduction in the amount of USP30 immunoprecipitated where **USP30-I-1** prevents HA-Ub-PA binding. The anti-HA blot shows efficient immunoprecipitation of HA-Ub-PA-labeled DUBs. (E) LC–MS/MS quantitation of the DUB-ABP complex immunoprecipitation shown in C. DUB activity is the intensity of the DUB in the presence of **USP30-I-1** normalized to the positive HA-Ub-PA control. (*n* = 2, ****p < 0.0001).

We performed ABPP-MS in SH-SY5Y neuroblastoma cell lysates to determine the potency and selectivity of **USP30-I-1**, a substituted cyanopyrrolidine derivative (Figure 1A). The ABP consists of HA-tagged ubiquitin with a propargylamine warhead (HA-Ub-PA) that reacts with the active site cysteine of cysteine protease DUBs, which forms the majority of the DUB family. Binding of HA-Ub-PA to a DUB can be detected via a ∼ 10 kDa mass shift of the DUB of interest by Western blot. Prevention of this binding by a small-molecule compound can inform on inhibitor potency, with densitometric analysis allowing for the extraction of IC_50_ values. Endogenous USP30 inhibition was confirmed via the prevention of HA-Ub-PA-USP30 binding by **USP30-I-1** in a concentration-dependent fashion (Figure 1B). **USP30-I-1** was observed to be a potent inhibitor of USP30, with an IC_50_ of 94 nM (Figure 1C). Immunoprecipitation of the HA tag on the HA-Ub-PA allows for DUB-ABP complexes to be purified and quantified by LC–MS/MS. The reduction in the presence of a DUB after treatment with a small-molecule compound is indicative of ABP binding prevention and, therefore, DUB inhibition, allowing for inhibitor selectivity to be assessed across the DUB family. Our quantitative MS analysis showcased the high selectivity of **USP30-I-1** for USP30, with no significant activity against any of the other 40 endogenous DUBs detected in SH-SY5Y extracts at the lowest inhibitor concentrations (Figure 1D,E). While there was a statistically significant reduction in JOSD2 with inhibitor treatment, the activity of JOSD2 was not reduced by more than 35% or in a concentration-dependent manner. Therefore, this may indicate unstable detection of the DUB by LC–MS/MS, rather than inhibition. At higher concentrations of 10 μM, however, we observed that **USP30-I-1** reduces the HA-Ub-PA labeling of a number of other DUBs (Figures S3, S4, and S5). This included approximately 50% inhibition of USP10. However, as USP10 is not strongly labeled by HA-Ub-PA, the inhibitory profile of USP10 by **USP30-I-1** could not be validated by the Western blot (Figure S6A). Moreover, **USP30-I-1** is far more potent for USP30 than it is for USP10 (Figure S6B). In conclusion, ABPP-MS demonstrated that **USP30-I-1** is a selective and potent inhibitor of USP30 at concentrations ≤1 μM.

**Figure 2:**
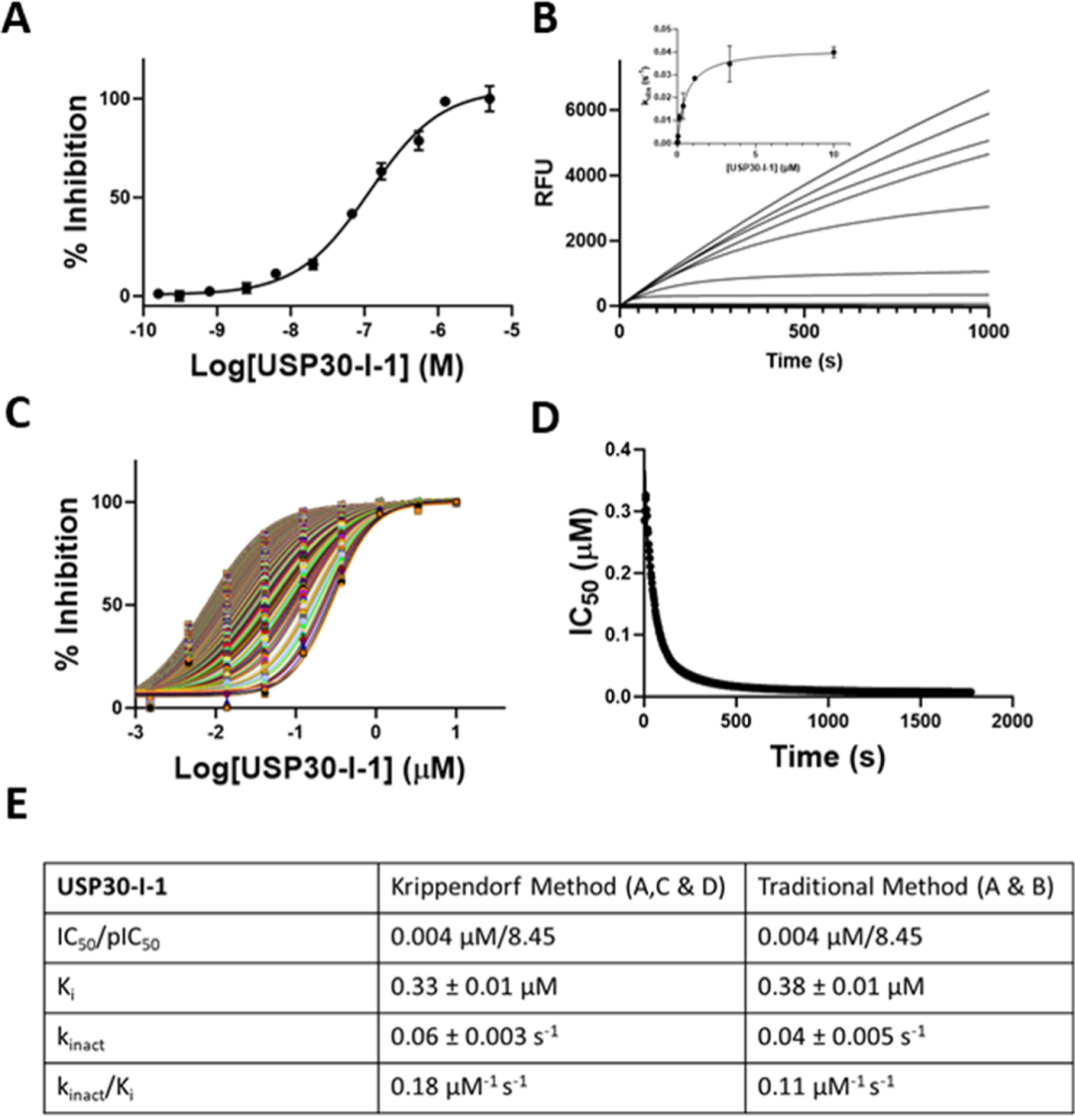
Covalent **USP30-I-1** tightly binds to recombinant USP30. (A) Dose-dependent inhibition of USP30 by **USP30-I-1**. (B) Progress curves recorded on the FLIPR Tetra. Traditional method for determining kinetic constants associated with covalent binders. *k*_obs,_ determined by fitting the progress curves to eq 2 (methods), is plotted vs [Compound], and fitted to eq 3 (methods) to determine *K*_I_ and *k*_inact_. (C) Krippendorf method used for determining covalent kinetic constants. Time-dependent IC_50_ curves. Each curve represents inhibition data at an individual incubation time from 3 to 600 s. (D) IC_50_ values vs incubation time fitted to eq 1 (see the methods section) to obtain *K*_I_ and *k*_inact_. (E) Data table of kinetic constants.

We then undertook in vitro biochemical assays to determine the full enzyme kinetics of **USP30-I-1**. In the first instance, fluorescence intensity measurements were used to monitor the interaction of a fluorogenic Ub-rhodamine substrate with a recombinant truncated version of USP30^36^ with and without the inhibitor. **USP30-I-1** inhibited USP30 in a dose-dependent manner, with an IC_50_ of ∼4 nΜ when USP30 was preincubated with inhibitors for 30 min (Figure 2A). The lower IC_50_ value observed for **USP30-I-1** in the in vitro work was likely a result of reduced nonspecific inhibitor occlusion as compared to the cellular matrix, and a similar phenomenon was observed in our **USP30**_**inh**_ work.^24^ Rates of inhibition determined from the Ub-rhodamine cleavage progress curves (Figure 2B) without USP30 preincubation were plotted against [**USP30-I-1**] (Figure 2B Inset) to determine *k*_inact_/*K*_I_ (traditional method). IC_50_ values were also measured and plotted against incubation time to calculate *k*_inact_/*K*_I_ (Krippendorf method) (Figure 2D,E), with both forms of analysis giving comparable parameters (Figure 2E). To conclude, **USP30-I-1** binds to USP30 in a tight manner and displays kinetic properties consistent with covalent attachment.

### 3.2 **USP30-I-1** Binds to the Catalytic Cysteine of USP30, Inducing Conformational Changes in the Active Site

**Figure 3:**
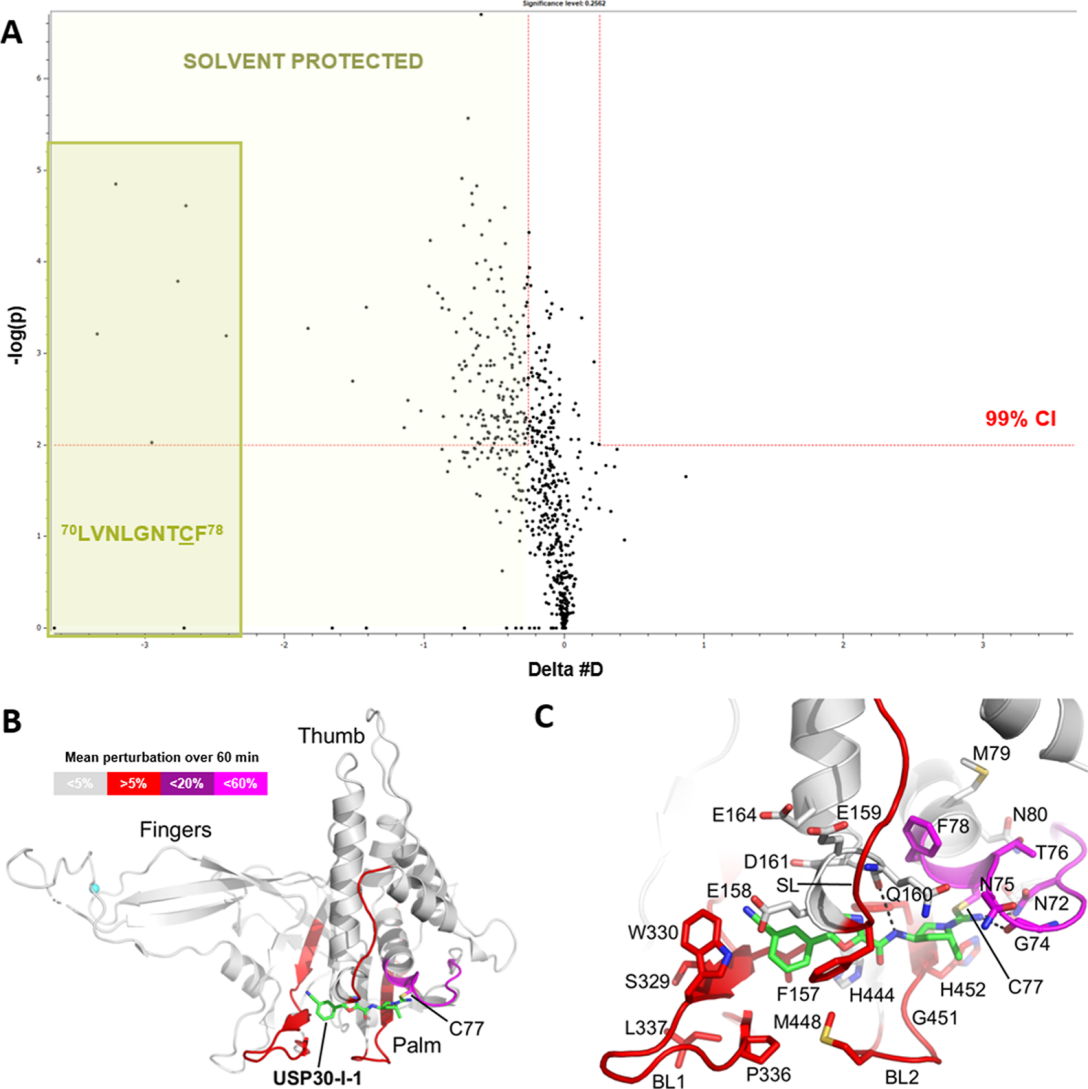
**USP30-I-1** binds to the catalytic cysteine of USP30. (A) HDX-MS shows that the USP30 region ^70^LVNLGNNTCF^78^ which surrounds the catalytic Cys77 (underlined) is significantly solvent protected in the presence of **USP30-I-1**. This implies that it is the primary location of compound binding and interaction with the protein. All labeling time points for the two peptides covering this region are shaded in dark green. (B) Modeled structure of human USP30 in complex with **USP30-I-1**. Structure of human USP30 catalytic domain highlighting the modeled position of **USP30-I-1** shown as a stick representation with carbon atoms colored green. The thumb, palm and fingers subdomains of the catalytic domain and the catalytic cysteine, Cys77, are highlighted. Regions identified in the HDX-MS analysis of USP30 in the presence of **USP30-I-1** are colored red (mean perturbation over 60 min of 5–20%) and magenta (mean perturbation over 60 min of <60%). (C). Close-up view of the putative **USP30-I-1** binding site highlighting flanking residues and key hydrogen-bonding interactions represented as dotted lines. The positions of blocking loop 1 (BL1), blocking loop 2 (BL2), and the switching loop (SL) are highlighted. Figure prepared using PyMOL (The PyMOL Molecular Graphics System, version 2.5.8; Schrödinger, LLC).

We used HDX-MS to determine the conformational dynamics associated with the interaction between **USP30-I-1** and USP30, in addition to pinpointing its precise location for small-molecule binding. From in excess of 700 unique peptide identifications, 161 peptides were used in the final data analysis, due to the ability to confidently detect each of them across all HDX-MS labeling time points (Tables S1 and S2).^37^ This resulted in an overall sequence coverage of 98% for the recombinant USP30 construct, which had an average redundancy of 4.71 peptides covering each amino acid. When directly comparing apo-USP30 to holo-USP30, the majority of the USP30 protein sequence had no significant differential deuterium uptake in the presence of **USP30-I-1**, confirming that, as one may expect upon interaction of a small-molecule with a much larger protein, binding was restricted to only a small portion of USP30 itself (Figures 3A and S7). The hybrid statistical analysis comparing the apo- and holo-states easily isolated a handful of peptides/labeling time points that became significantly shielded from the deuterium buffer in the presence of the compound, indicative of binding and/or interaction. These primarily matched to peptides covering the sequence ^70^LVNLGNTCF^78^, which comprises the catalytic Cys77 and the preceding loop and where a mean perturbation in deuterium labeling between states of up to 60% was observed across all time points. It should be noted, however, that solely in the holo-state, the overall peptide quality was reduced in this region of USP30, presumed to a direct result of the strong covalent attachment of the inhibitor to the peptide under HDX-MS experimental conditions. This peptide contains the catalytic Cys77 covalently modified with the inhibitor and may therefore have different ionization properties, while the covalent modification may result in changes (albeit comparatively very small) to the deuteration properties of this region. Taking all of this into consideration, the true perturbation in deuteration of peptides covering this region may be less than what is observed by our measurements and does not detract from our primary goal of identifying which regions of USP30 are important for its inhibition.

Nevertheless, these observations provide an additional layer of confidence that catalytic Cys77 is indeed the primary binding site of **USP30-I-1** on the protein. We subsequently mapped all HDX-MS behaviors onto the structure of human USP30 catalytic domain using the X-ray structure of human USP30 catalytic domain in complex with the covalent inhibitor, 552, and a Fab fragment antibody, in which the covalent inhibitor and Fab fragment antibody had been removed, as the template (Figure 3B). This structure is representative of the catalytic domain conformation in complex with a covalent inhibitor, as exemplified by both covalent inhibitor structures present in the protein data bank. Our mapping further highlighted the strong interaction of **USP30-I-1** with the region encompassing the catalytic Cys77 (highlighted in magenta in Figure 3B) but also supported the identification of other regions of human USP30 catalytic domain (highlighted in red) that become solvent protected, albeit to a much lesser extent. These include the sequences ^150^YRWQISSF^157^ (corresponding to the switching loop), ^322^CIHLQRLSWSSHGTPLKRH^340^ (corresponding to blocking loop 1), ^446^GDMHSGHFVTY^456^ (corresponding to blocking loop 2), and ^465^NPLSTSNQWL^474^ (residues preceding and forming part of the β-strand that accommodates catalytic residue, Ser477, in the central β-sheet in the palm subdomain) (Figure 3B,C). In conclusion, our differential HDX-MS allowed us to pinpoint regions of USP30 that are involved in **USP30-I-1** binding.

**Figure 4:**
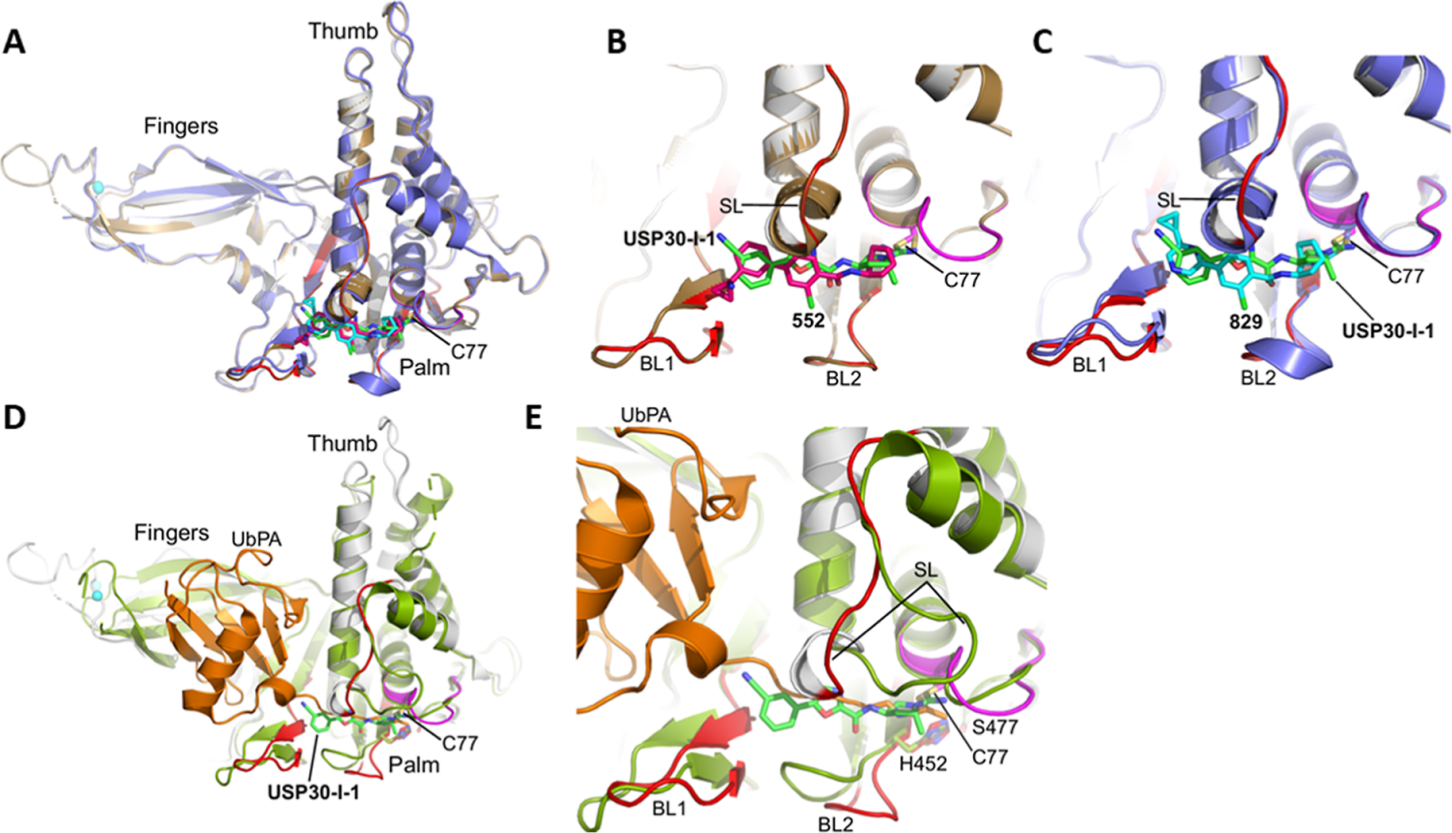
Comparison of **USP30-I-1** binding to USP30 with Ub and analogous covalent USP30 inhibitors. (A). Superposition of the modeled structure of human USP30 in complex with **USP30-I-1** on the X-ray structures of human USP30 in complex with the covalent inhibitors, 552 (PDB code: 8D1T) and 829 (PDB code: 8D0A). The thumb, palm and fingers subdomains of the catalytic domain and the catalytic cysteine, Cys77, are highlighted. The catalytic domain of the modeled structure of human USP30 in complex with **USP30-I-1** is colored gray with regions implicated in compound binding from the HDX-MS analysis colored red (mean perturbation over 60 min of 5–20%) and magenta (mean perturbation over 60 min of <60%). **USP30-I-1** (carbon atoms in green), 552 (carbon atoms in hot pink), and 829 (carbon atoms in cyan) are shown as stick representations. The catalytic domain of USP30 in complex with 552 and 829 are colored brown and violet, respectively. The modeled pose of **USP30-I-1** correlates well with 552 and 829. (B). Close-up view of the superimposed structure of human USP30 in complex with 552 (carbon atoms in hot pink) with the modeled structure of **USP30-I-1** (carbon atoms in green). The positions of BL1, BL2, and SL are highlighted. (C). Close-up view of the superimposed structure of human USP30 in complex with 829 (carbon atoms in cyan) with the modeled structure of **USP30-I-1** (carbon atoms in green). The positions of BL1, BL2, and SL are highlighted. (D). Superposition of the modeled structure of human USP30 in complex with **USP30-I-1** on the X-ray structure of human USP30 in complex with ubiquitin-propargylamide (UbPA; PDB code: 5OHK). The catalytic domain of the modeled structure of human USP30 in complex with **USP30-I-1** is colored gray with regions implicated in compound binding from HDX-MS analysis are colored as above. **USP30-I-1** is shown with carbon atoms colored green. The catalytic domain of USP30 in complex with UbPA is colored lime with UbPA shown in orange. **USP30-I-1** is predicted to bind in the thumb-palm cleft that guides the ubiquitin C-terminus into the active site. (E). Close-up view of the superimposed structures of human USP30 in complex with UbPA and the modeled structure of **USP30-I-1** (carbon atoms in green). Catalytic triad residues (C77, H452 and S477) are highlighted and shown as stick representations. Figure prepared using PyMOL (The PyMOL Molecular Graphics System, version 2.5.8; Schrödinger, LLC).

Finally, we performed molecular docking studies to computationally validate our *in vitro* work. As illustrated in the pose displayed in Figure 3B,C, **USP30-I-1** is predicted to bind in the thumb-palm cleft that guides the ubiquitin C-terminus into the active site. **USP30-I-1** forms a thioimidate with the catalytic cysteine Cys77, with the imine moiety accepting a hydrogen bond from the side chain of Asn72 and acting as a hydrogen bond donor to the main chain carbonyl of Gly74. In addition, the amide moiety of **USP30-I-1** hydrogen bonds to the main chain carbonyl of Gln160 (Figure 3C). The docking pose for **USP30-I-1** correlates perfectly with our HDX-MS results with both suggesting that the USP30 catalytic Cys77 is the primary binding site of **USP30-I-1**. In addition, the docking pose of **USP30-I-1** correlates well with the X-ray structures of human USP30 catalytic domain in complex with the covalent inhibitors, 552 (PDB code: 8D1T; unpublished) and 829 (PDB code: 8D0A; unpublished) (Figure 4A,B,C). In the complexes of USP30 with 552 and 829, the switching loop adopts an “in” conformation, whereas in the complex with ubiquitin-propargylamide (PDB code: 5OHK) it^36^ adopts an “out” conformation, which, coupled with conformational differences in blocking loops 1 and 2, allow the C-terminal tail of the ubiquitin substrate to be accommodated in the thumb-palm cleft leading to active sites. Hence, the predicted binding site of **USP30-I-1** based on our modeling studies would sterically clash with the C-terminal tail of ubiquitin (Figure 4D,E).

**Figure 5:**
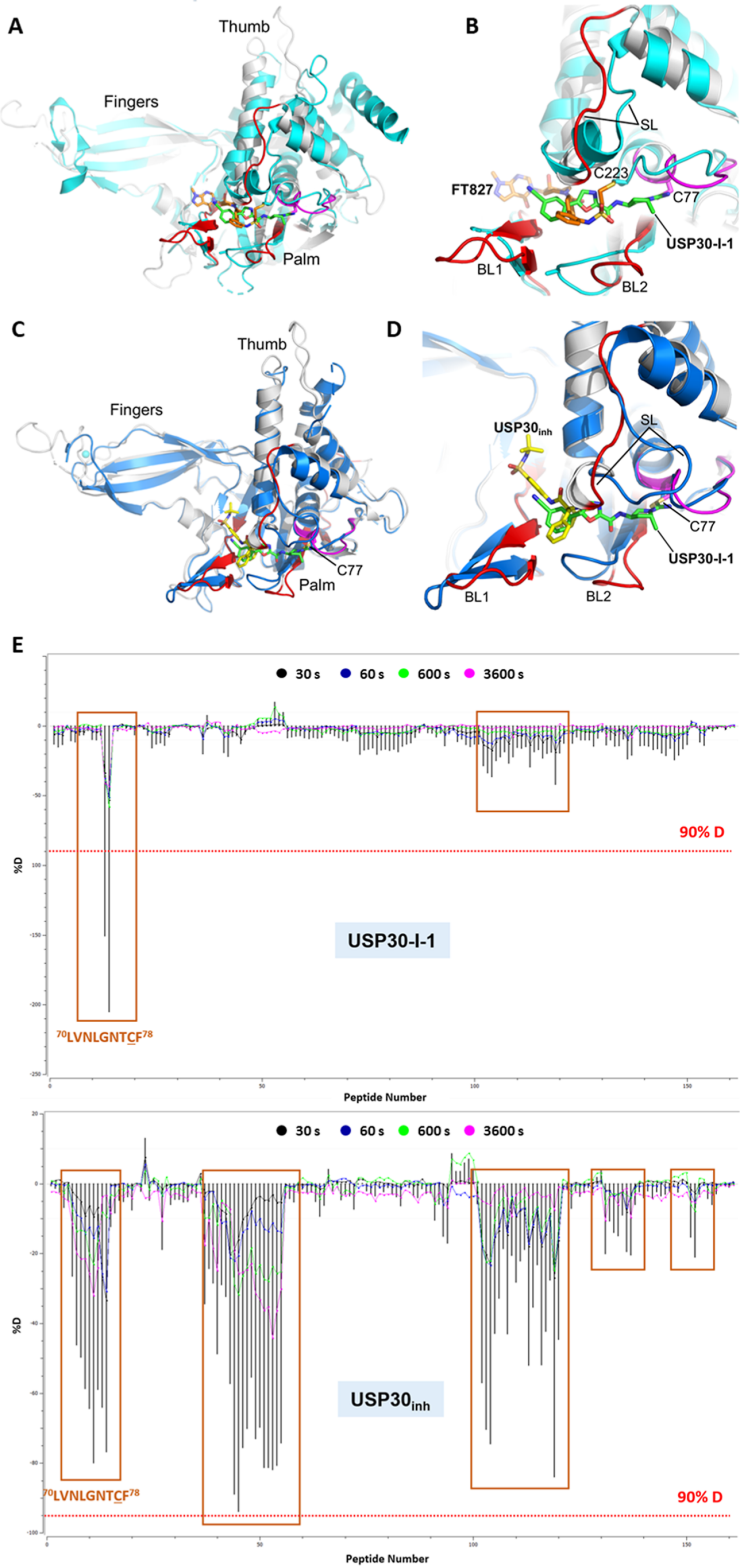
Comparison of covalent and noncovalent inhibitor binding in USP7 and USP30. (A) Superposition of the modeled structure of human USP30 in complex with **USP30-I-1** on the X-ray structure of human USP7 in complex with the covalent inhibitor, FT827 (PDB code: 5NGF; r.m.s.d. = 2.5 Å, 249 residues aligned). (B) Close-up view of the superimposed modeled structure of human USP30 in complex with **USP30-I-1** (green carbon atoms) on the X-ray structure of human USP7 in complex with the covalent inhibitor, FT827 (orange carbon atoms). The positions of BL1, BL2, SL, and the catalytic cysteines (Cys77 in USP30 and Cys223 in USP7) are highlighted. The Cαs of Cys77 and Cys223 are separated by approximately 5.2 Å, which, combined with differences in the conformations of BL1, BL2, and SL, result in FT827 extending closer toward the fingers subdomain than **USP30-I-1**. (C) Superposition of the modeled structures of human USP30 in complex with **USP30-I-1** and **USP30**_**inh**_.^24^ The catalytic domain of the modeled structure of human USP30 in complex with **USP30-I-1** is colored gray with regions implicated in compound binding from HDX-MS analysis colored red (mean perturbation over 60 min of 5–20%) and magenta (mean perturbation over 60 min of <60%). **USP30-I-1** is shown with carbon atoms colored green. The catalytic domain of the modeled structure of human USP30 in complex with **USP30**_**inh**_ is colored blue. **USP30**_**inh**_ is shown with carbon atoms colored yellow. (D) Close-up view of the superimposed modeled structures of human USP30 in complex with **USP30-I-1** (carbon atoms in green) and **USP30**_**inh**_ (yellow carbon atoms). The positions of BL1, BL2, and SL are highlighted. Both inhibitors are predicted to bind within the thumb-palm cleft with **USP30**_**inh**_ residing approximately 7.9 Å away from Cys77 and extending out toward the fingers subdomain. (E) HDX-MS residual plot of USP30 in complex with **USP30-I-1** and **USP30**_**inh**_. A greater overall solvent protection is observed for USP30 in the presence of the noncovalent inhibitor, as compared to its covalent counterpart. A cutoff of 90% D perturbation was used for ease of comparison, which is the highest value observed for the **USP30**_**inh**_ study and common to both experiments. **USP30-I-1** primarily induces solvent protection in the region encompassing the catalytic Cys77, whereas the noncovalent **USP30**_**inh**_ results in smaller HDX-MS perturbations, albeit, extended to several regions of the protein. Figure prepared using PyMOL (The PyMOL Molecular Graphics System, version 2.5.8; Schrödinger, LLC).

Distinct apo-form and ubiquitin-bound conformational states have been observed for other USP family members and are implicated in the catalytic cycle.^38,39^ In addition, several USP inhibitors have been shown to preferentially target the apo-form conformation. For example, the noncovalent USP7 inhibitor, FT671 (PDB code: 5NGE), and covalent USP7 inhibitor, FT827 (PDB code: 5NGF), specifically target the catalytically incompetent apo-form state in which the switching loop adopts an “in” conformation.^40^ A comparison of **USP30-I-1** with FT827 reveals that the binding site is broadly similar but that there are conformational differences in the region accommodating the catalytic cysteine, the switching loop, and blocking loops 1 and 2 (Figure 5A,B). These conformational differences result in FT827 extending closer toward the finger subdomain than the modeled position of **USP30-I-1**. We envisage that USP30 may also adopt distinct conformational states during its catalytic cycle, which can be exploited by inhibitors, but we await the determination of an experimental structure of the apo-form USP30 catalytic domain to confirm our hypothesis.

There are 26 residues with an atom residing within 5 Å of the predicted **USP30-I-1** binding site (Figure 3B). Inhibitor selectivity is likely to be conferred by sequence substitutions in these residues compared with other USP family members coupled with conformational differences in the regions flanking the proposed **USP30-I-1** binding site, including the switching and blocking loops. In summary, our molecular docking analysis identified residues implicated in **USP30-I-1** binding, which is in perfect agreement with those identified through differential HDX-MS.

### 3.3 Comparison of USP30 Inhibition by Covalent Cyanopyrrolidine **USP30-I-1** and Noncovalent Benzosulfonamide **USP30**_**inh**_ Compound

We previously performed biochemical, kinetic, and structural characterization of USP30 in complex with a noncovalent small-molecule benzosulfonamide **USP30**_**inh**_.^24^ By directly comparing our two studies, we could decipher commonalities and differences between covalent and noncovalent mechanisms of USP30 inhibition in terms of the kinetics of complex formation and structural dynamics.

For covalent compounds, IC_50_ is a poor measure of potency, as it is a time-dependent process and often correlates poorly with efficacy. Determination of *k*_inact_/*K*_I_ is the gold standard for assessing the potency of covalent inhibitors. *k*_inact_/*K*_I_ is a second-order rate constant, which defines the efficiency of covalent bond formation by incorporating the affinity of the initial reversible binding of the inhibitor (*K*_I_) and the rate at which the enzyme is inactivated by covalent bond formation (*k*_inact_) with a higher value corresponding to a more potent inhibitor. **USP30-I-1** shows both a high initial binding affinity (*K*_I_ ∼ 350 nM) and a fast rate of specific inactivation (*k*_inact_ ∼ 0.15 s^–1^). Both **USP30-I-1** and noncovalent **USP30**_**inh**_ show time-dependent inhibition, with **USP30**_**inh**_ showing two-step, slow, and tight binding kinetic behavior consistent with a covalent inhibitor. The noncovalent inhibitor **USP30**_**inh**_ acts via a mechanism similar to that of a covalent inhibitor. As described previously,^24^ a two-step inhibition process was observed leading to time-dependent inhibition, the second step can be defined by an on (*k*_5_) and off (*k*_6_) rate. In the case of **USP30**_**inh**_, *k*_6_ can be calculated as being essentially zero, this simplifies the equation and *k*_5_ is, therefore, directly comparable to kin_act_ derived for covalent compounds. The second step of inhibition by **USP30**_**inh**_ is essentially irreversible (*k*_6_ = 0.00033 s^–1^) allowing us to compare *k*_5_/*K*_iapp_ for **USP30**_**inh**_ with *k*_inact_/*K*_I_ for **USP30-I-1** as a measure of potency. Using these values, the compounds have a similar potency (0.18 and 0.20 μM^–1^ s^–1^ for **USP30-I-1** and **USP30**_**inh**_, respectively), in the case of **USP30**_**inh**_, the potency is driven by the rate at which the irreversible complex forms with the initial affinity being lower than that of **USP30-I-1** (1.27 μM and 350 nM for **USP30-I-1** and **USP30**_**inh**_, respectively). It will be interesting to see whether these differences have an effect on the cellular potency.

Our previous study also employed HDX-MS and computational docking to elucidate the molecular architecture and geometry of USP30 complex formation with the noncovalent inhibitor, **USP30**_**inh**_.^24^ Both compounds interact with the catalytic Cys77, but **USP30-I-1** induces a higher degree of solvent protection than does **USP30**_**inh**_. Only the catalytic region undergoes substantial perturbation in the presence of the covalent compound, suggesting that it is a more targeted interaction. A much larger area is perturbed in the presence of the noncovalent inhibitor, implying that the compound is moving about and reorienting itself within the binding pocket (Figures S8 and 5E). **USP30**_**inh**_ is also predicted to bind to the thumb-palm cleft of the catalytic domain. However, compared with **USP30-I-1**, which forms a covalent adduct with the catalytic Cys77, the modeled position of **USP30**_**inh**_ resides approximately 7.9 Å away at its closest point from the thiol side chain of this cysteine (Figure 5C,D). The X-ray structure of human USP30 catalytic domain in complex with UbPA (PDB code: 5OHK; Gersch et al., 2017^36^) was used as the target receptor for **USP30**_**inh**_ because there were no inhibitor-bound structures available at that time, which results in differences in conformation in the switching and blocking loop regions compared with the structure of USP30 in complex with the covalent inhibitor, 552 (PDB code: 8D1T), that was used for **USP30-I-1** docking studies. However, we note that the docking pose of **USP30**_**inh**_ correlated well with the HDX-MS data and is predicted to bind to an equivalent site as the noncovalent USP7 inhibitor, FT671, which resides approximately 5 Å away from the catalytic cysteine (Turnbull et al., 2017^40^). Hence, while both studies indicate that **USP30**_**inh**_ and **USP30-I-1** are likely to reside within the thumb-palm cleft, differences in their HDX-MS profiles suggest that there are differences in their binding modes, which places **USP30**_**inh**_ closer toward the fingers subdomain compared with the predicted binding site for **USP30-I-1** (Figure 5E).

Very recently, the X-ray structure of a human USP30 chimera that features the fingers of USP14 and the USP35 box 4/5 insertion (USP30^ch3^), has been determined in complex with the noncovalent inhibitor, NK036.^41^ NK036 binds in a cryptic pocket, which is revealed by a large inhibitor-induced conformation change in the switching loop that has not been observed in other reported ubiquitin propargylamide-bound (Ub-PA; PDB codes: 5OHN and 5OHK) or covalent inhibitor-bound (PDB codes: 8D1T and 8D0A) USP30 structures. NK036 represents a solubility-enhanced derivative of a benzosulfonamide-containing inhibitor, **USP30**_**inh**_, that was previously docked into the structure of human USP30 catalytic domain taken from its complex with Ub-PA (PDB code: 5OHK; O’Brien et al., 2023^24^). Docking predicts that the fluorophenyl moiety of **USP30**_**inh**_ will sterically clash with Ub Leu73 when compared with the structure of the USP30-Ub-PA complex, in an identical manner to the equivalent moiety in NK036 in the USP30^ch3^-NK036 complex. Furthermore, the central phenyl ring of **USP30**_**inh**_ is predicted to bind close to Leu328, Met448, and His449, similarly to NK036. In contrast, the predicted and experimental binding modes of the benzosulfonamide moieties of **USP30**_**inh**_ and NK036 are strikingly different, respectively, extending out toward the fingers subdomain or residing in a cryptic pocket. These differences arise from the unanticipated switching loop conformation observed in the USP30^ch3^-NK036 complex, which is unaccounted for in the **USP30**_**inh**_ docking study that used the conformation of USP30 in its complex with Ub-PA as the template. Given the similarities between NK036 and **USP30**_**inh**_, the binding modes of both inhibitors are anticipated to be the same. In the current study, the inhibitor, **USP30-I-1**, forms a covalent adduct with the catalytic cysteine, Cys77, and is predicted to reside in the thumb-palm cleft that guides the ubiquitin C-terminus into the active site. We are confident in the predicted binding mode of **USP30-I-1** since it correlates well with the HDX-MS data presented in this paper. In addition, the docking pose of **USP30-I-1** agrees with the experimental structures of USP30 in complex with the covalent inhibitors, 552 (PDB code: 8D1T; unpublished) and 829 (PDB code: 8D0A; unpublished), in which the switching loop adopts an “in” conformation, suggesting that it is unlikely **USP30-I-1** will occupy a cryptic pocket. However, an experimental structure of USP30 in complex with **USP30-I-1** would be required to validate the docking results.

## 4 Conclusions

**USP30-I-1** represents a covalent small-molecule cyanopyrrolidine inhibitor that exhibits high potency and selectivity for the mitochondrial DUB, USP30. HDXMS studies reveal that covalent attachment of **USP30-I-1** to Cys77 results in significant solvent protection in the region that flanks the catalytic cysteine, whereas less noticeable structural and conformational changes are seen in other regions. Inhibitor binding blocks the interaction of USP30 with Ub substrate molecules, preventing isopeptide bond cleavage. This enhanced understanding of the molecular mechanisms surrounding USP30 inhibition will aid in the creation of next-generation inhibitors that target neurodegenerative and cardiovascular diseases.

## Data Availability

All ABPP-MS proteomics raw files have been deposited to the ProteomeXchange Consortium and can be accessed through the identifier PXD054041. Similarly, HDX-MS data can be downloaded using PXD054057.

## Supporting Information Available

Further description to support enzyme kinetics observations, purity profile of **USP30-I-1**, confirmation of covalent USP30 complex formation with **USP30-I-1** by RapidFire MS. , ABPP-MS analysis of **USP30-I-1** for inhibiting USP30. LC–MS/MS quantitation of HA-Ub-PA enriched DUBs with **USP30-I-1** at 0 and 10 mM relative to positive control (*****p* < 0.0001), uncropped blots of ABPP-MS analysis of **USP30-I-1** for inhibiting USP30, uncropped ABPP-MS blots for USP30, HA, and β-actin and Uncropped blots used in the analysis of LC–MS/MS quantitation of HA-Ub-PA enriched DUBs with **USP30-I-1** at 0 and 10 μΜ relative to positive control (*****p* < 0.0001). USP30, HA, and β-actin blots are shown, comparison of ABPP-MS of **USP30-I-1** for inhibiting USP30 and USP10, HDX-MS Woods plot of USP30 in complex with **USP30-I-1**, and HDX-MS volcano plots of USP30 in complex with **USP30-I-1** and **USP30inh** (PDF)
HDX summary table^37^ (XLSX)
HDX data table^37^ (XLSX)

## Abbreviations

ABPP-MS: activity-based protein profiling mass spectrometry
DIA: data-independent acquisition
DMSO: dimethyl sulfoxide
DUB: Deubiquitinase
HDX-MS: Hydrogen–Deuterium eXchange Mass Spectrometry
LFQ: label-free quantitation
MOM: mitochondrial outer membrane
PD: Parkinson’s disease
PDB: protein data bank
SAR: structure activity relationship
Ub: ubiquitin
UPS: ubiquitin proteasome system
USP30: ubiquitin specific protease 30
**USP30-I-1**: covalent inhibitor of USP30
