# Supplementary material for: Structural Dynamics of the Ubiquitin Specific Protease USP30 in Complex with a Cyanopyrrolidine-Containing Covalent Inhibitor
Source: J Proteome Res. 2025 Jan 13;24(2):479–90. doi: 10.1021/acs.jproteome.4c00618 (PMC11812085; doi:10.1021/acs.jproteome.4c00618)
Supplement: Supplementary file 1 — pr4c00618_si_001.pdf [file pr4c00618_si_001.pdf]

Structural Dynamics of the Ubiquitin Specific Protease USP30 in Complex with a Cyanopyrrolidine-Containing Covalent Inhibitor

Darragh P O'Brien[1*], Hannah BL Jones[1], Yuqi Shi[2], Franziska Guenther[3], Iolanda Vendrell[1,4], Rosa Viner[2], Paul E Brennan[3], Emma Mead[3], Tryfon Zarganes-Tzitzikas[3], John B Davis[3], Adán Pinto-Fernández[1,4], Katherine S England[3], Emma J Murphy[3], Andrew P Turnbull[5*], and Benedikt M Kessler[1,4*]

[1]Target Discovery Institute, Centre for Medicines Discovery, Nuffield Department of Medicine, University of Oxford, OX3 7FZ, UK

[2]Thermo Fisher Scientific, San Jose, California, CA 95134, USA

[3]ARUK-Oxford Drug Discovery Institute, Centre for Medicines Discovery, Nuffield Department of Medicine, University of Oxford, OX3 7FZ, UK

[4]Chinese Academy of Medical Sciences Oxford Institute, Nuffield Department of Medicine, University of Oxford, OX3 7BN, UK

[5]Cancer Research Horizons, Francis Crick Institute, London, NW1 1AT, UK

*Corresponding authors:
darragh.obrien@ndm.ox.ac.uk
andrew.turnbull@cancer.org.uk
benedikt.kessler@ndm.ox.ac.uk

SUPPORTING INFORMATION TABLE OF CONTENTS

Text S1. Enzyme Kinetics

Figure S1. Purity profile of USP30-I-1.

Figure S2. Confirmation of covalent USP30 complex formation with USP30-I-1 by RapidFire MS.

Figure S3. ABPP-MS analysis of USP30-I-1 for inhibiting USP30.

Figure S4. Uncropped blots of ABPP-MS analysis of USP30-I-1 for inhibiting USP30.

Figure S5. Uncropped ABPP-MS blots for USP30, HA, and β-actin.

Figure S6. Comparison of ABPP-MS of USP30-I-1 for inhibiting USP30 and USP10.

Figure S7. HDX-MS Woods plot of USP30 in complex with USP30-I-1.

Figure S8. HDX-MS Volcano Plots of USP30 in complex with USP30-I-1 and $USP30_{inh}$.

Enzyme Kinetics

The traditional method to determine the kinetics of a covalent compound was also used. Progress curves were fitted to Eq.2, where [P] is product formed, *t* is time, v_i is the initial rate of the reaction and k_{obs} the rate at which the system is inactivated.

$$[P] = \frac{v_i}{k_{obs}}[1 - exp(-k_{obs}t)]$$

Eq.2

The resulting k_{obs} values were plotted against concentration and fitted to Eq.3a to obtain K_I and k_{inact}.

$$k_{obs} = k_{\mathrm{inact}}\left(\frac{[I]}{K_I + [I]}\right)$$

Eq.3

Text S1. Enzyme Kinetics.

FractionLynx Report - Page 2

Sample: 2 Vial:5,3:5,H ID:

File: Date:20-Oct-2023 Time:16:24:32

Description:

Printed: Wed Nov 01 16:01:49 2023

Sample Report (continued):

UV Detector: 254 Smooth (SG, 1x1) 2.499e-1

Range: 3.11e-1

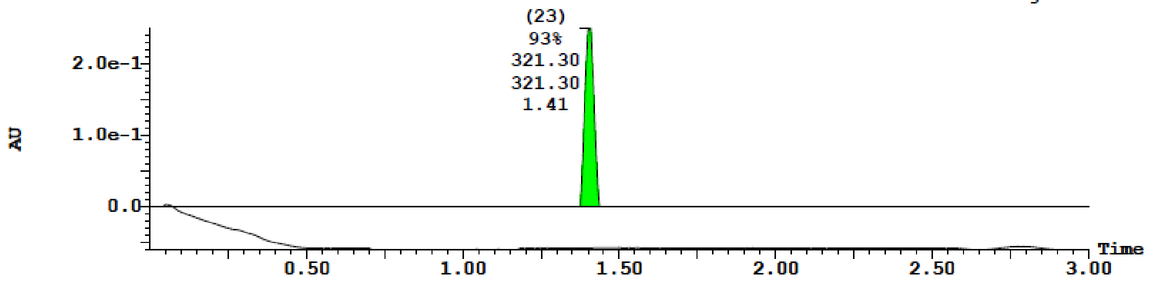

Peak Number	Compound	Time	AreaAbs	Area %Total	Width	Height	Mass Found
23	Found	1.41	1e+004	93.41	0	3e+005	321.30, 321.30
26		1.71	9e+002	6.59	0	2e+004	

(2) ELSD Signal Smooth (SG, 1x1) 670.894

Range: 671.639

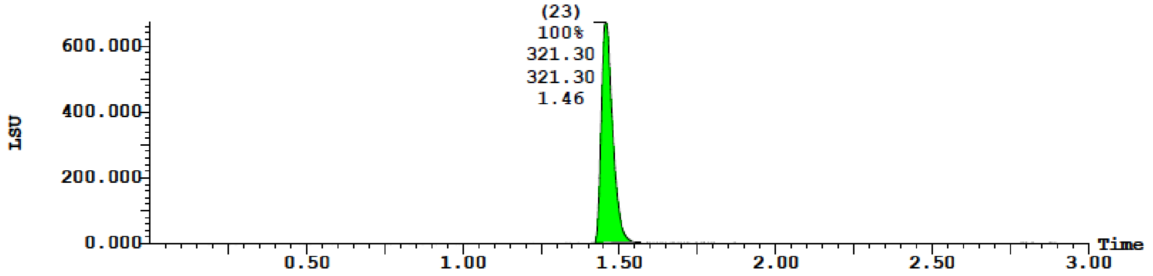

Peak Number	Compound	Time	AreaAbs	Area %Total	Width	Height	Mass Found
23	Found	1.46	3e+004	100.00	0	7e+005	321.30, 321.30

Figure S1. Purity profile of USP30-I-1. The compound was deemed 100% pure by HPLC analysis and measurement at 254 nm.

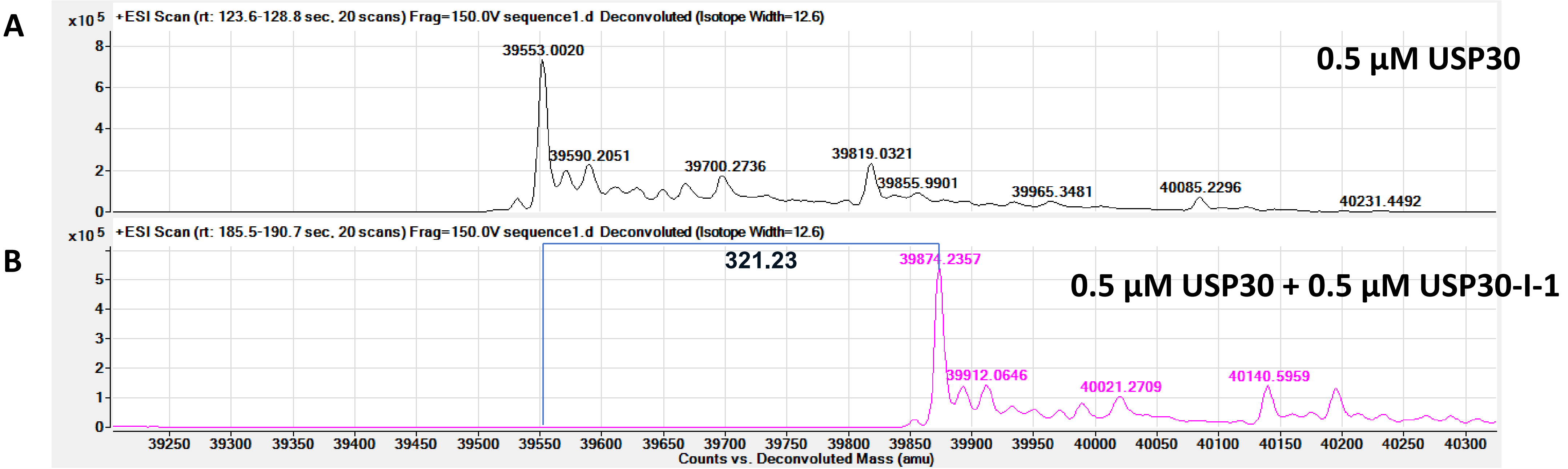

Figure S2. Confirmation of covalent USP30 complex formation with USP30-I-1 by RapidFire MS. A. 0.5 µM USP30 shows a peak at the expected MW of 39552.24 **B.** After a 300 s incubation at room temperature with 0.5 µM **USP30-I-1** the peak is shifted by 321.23 showing the formation of a covalent adduct.

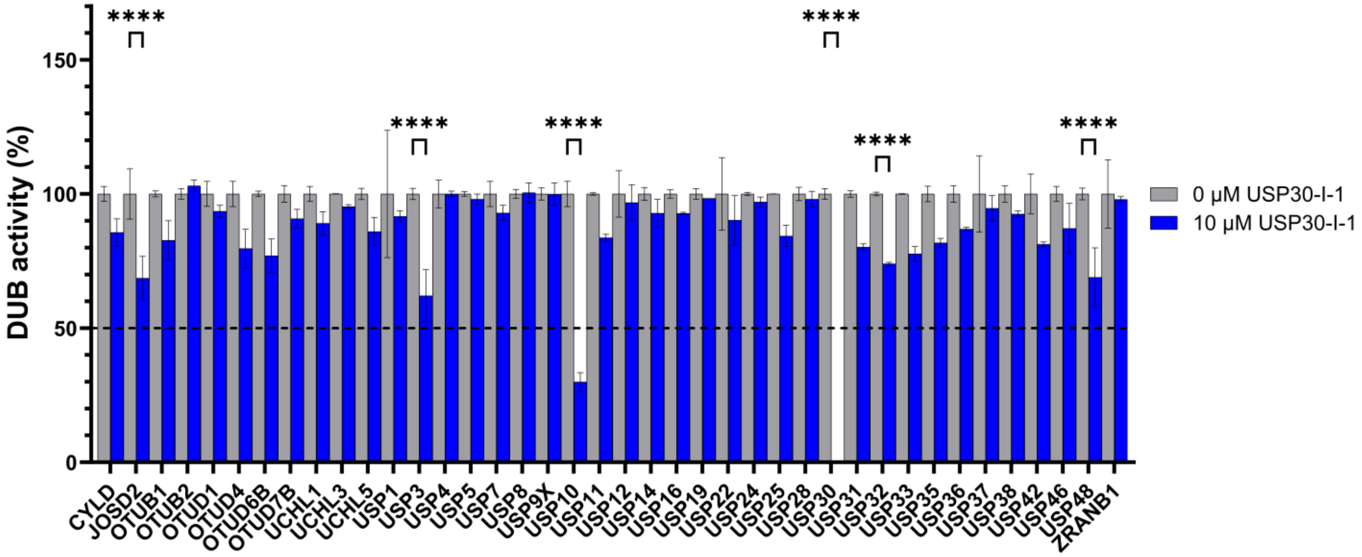

Figure S3. ABPP-MS analysis of USP30-I-1 for inhibiting USP30. A. LC-MS/MS quantitation of HA-Ub-PA enriched DUBs with **USP30-I-1** at 0 and 10 µM relative to positive control (****$p < 0.0001$).

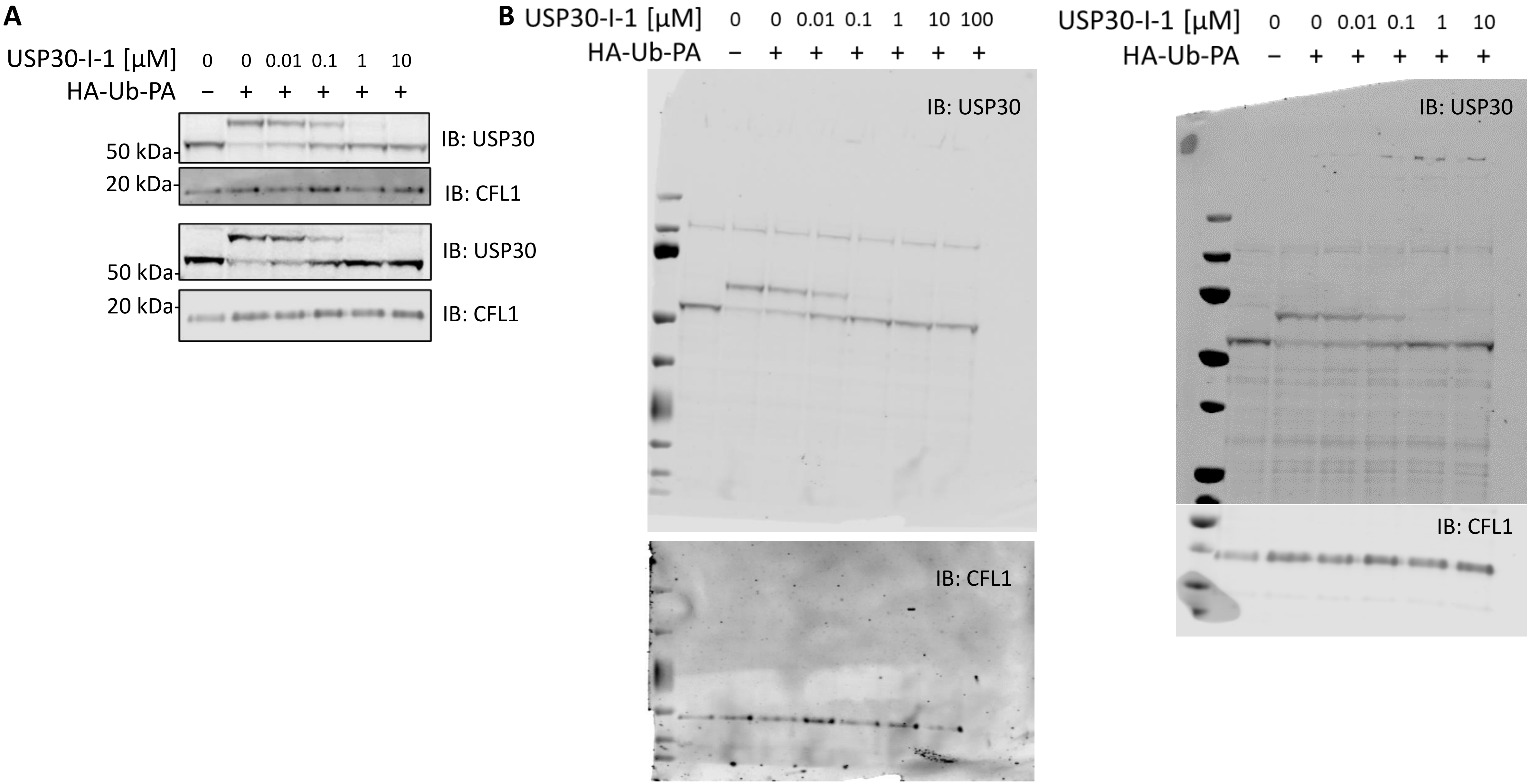

Figure S4. Uncropped blots of ABPP-MS analysis of USP30-I-1 for inhibiting USP30. (A) LC-MS/MS quantitation of HA-Ub-PA enriched DUBs with **USP30-I-1** at 0 and 10 µM relative to positive control (****$p < 0.0001$). **(B)** Uncropped blots of USP30 and CFL1 used in the analysis.

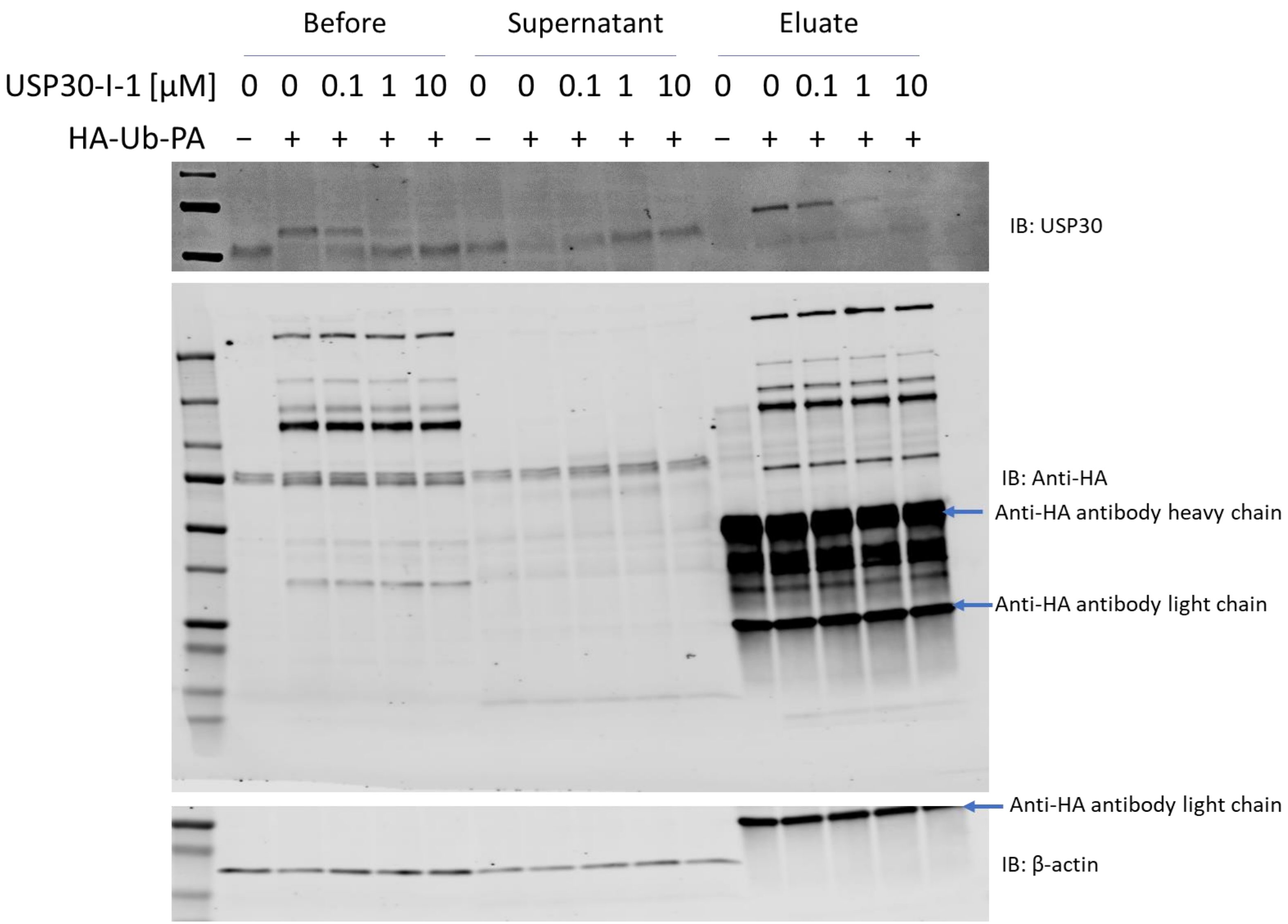

Figure S5. Uncropped ABPP-MS blots for USP30, HA, and β-actin **.** Uncropped blots used in the analysis of LC-MS/MS quantitation of HA-Ub-PA enriched DUBs with **USP30-I-1** at 0 and 10 μM relative to positive control (****$p < 0.0001$). USP30, HA, and β-actin blots are shown.

A

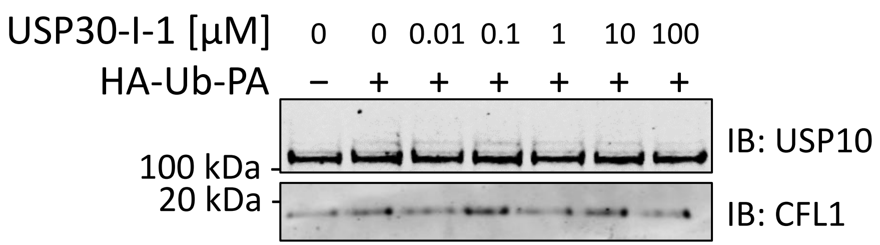

*USP10 ABP labelling not seen strongly – can be increased by glucose starvation, but still not good probe labelling seen (Fig 5B): https://www.ncbi.nlm.nih.gov/pmc/articles/PMC4836875/

B

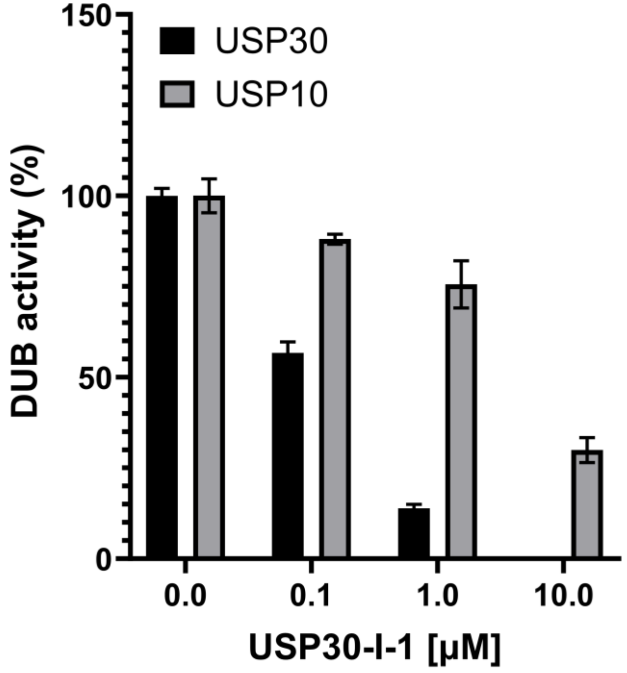

Figure S6. Comparison of ABPP-MS of USP30-I-1 for inhibiting USP30 and USP10. A. No strong HA-Ub-PA labelling of USP10. **B.** LC-MS/MS quantitation of HA-Ub-PA enriched USP30 & USP10, demonstrating higher potency of USP30-I-1 for USP30 inhibition compared with USP10.

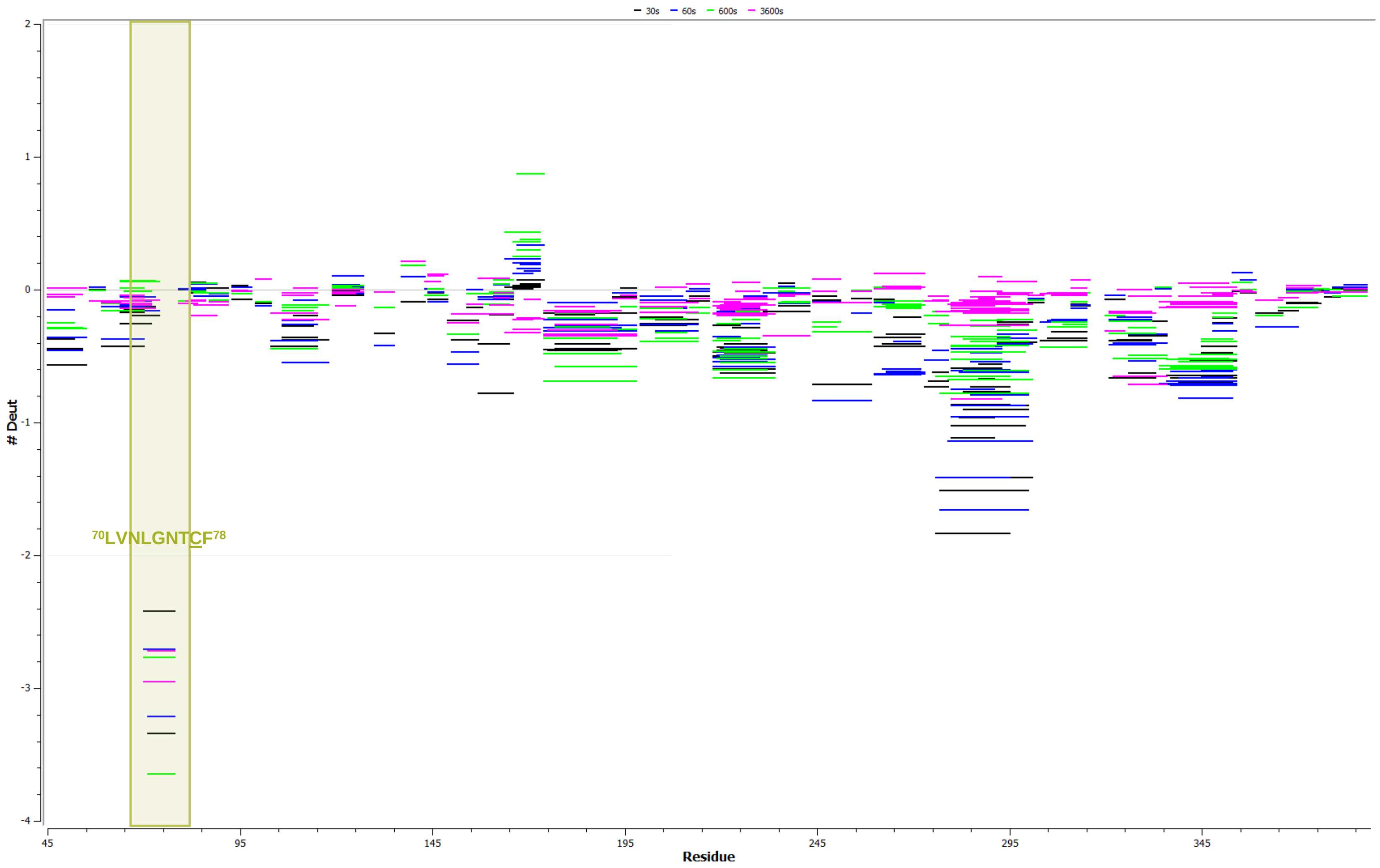

Figure S7. HDX-MS Woods plot of USP30 in complex with USP30-I-1. A greater overall solvent protection is observed for USP30 in the presence of the non-covalent inhibitor, as compared to its covalent counterpart. **USP30-I-1** primarily induces solvent protection in the region encompassing the catalytic Cys77, as highlighted in the plot.

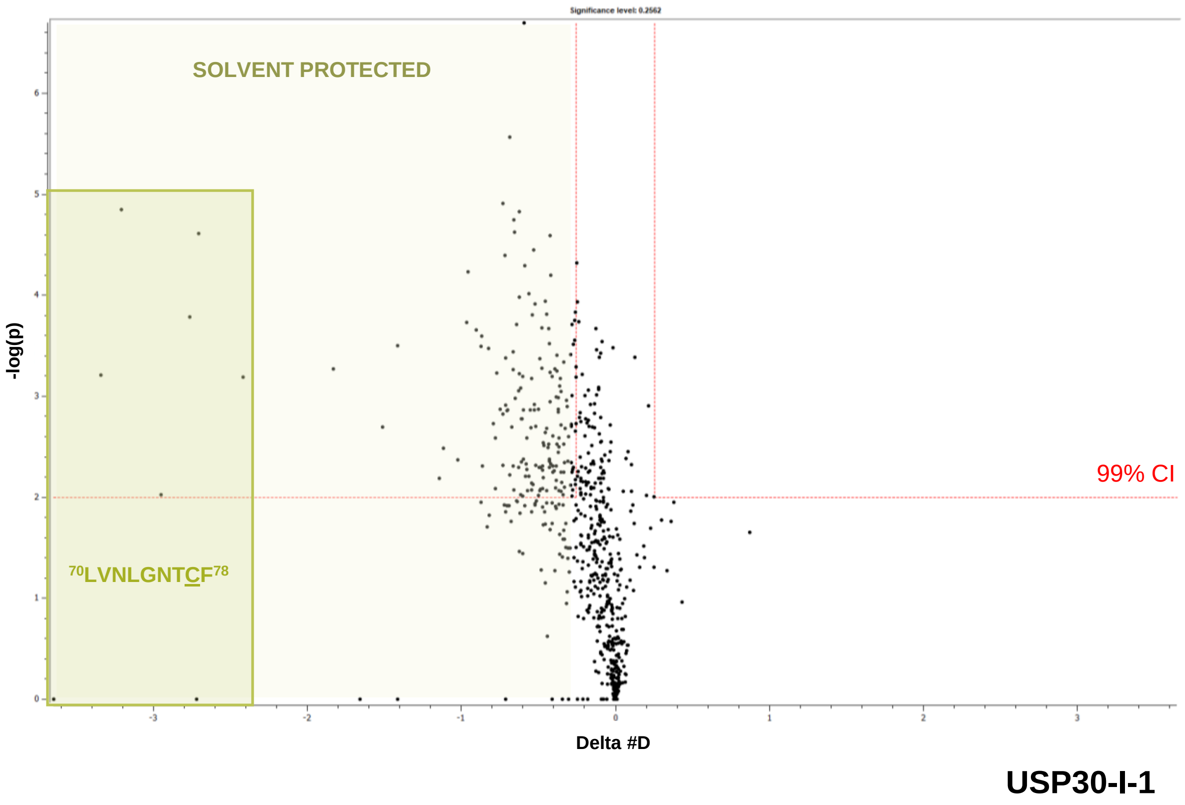

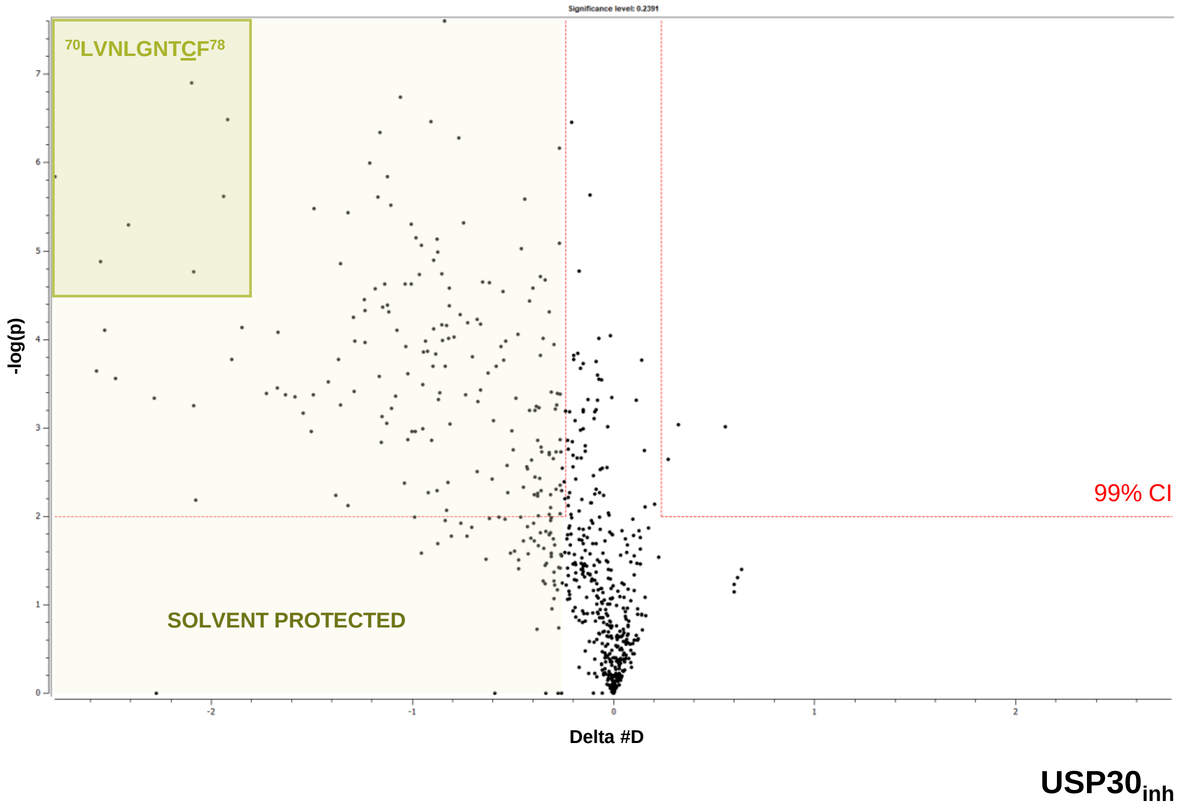

Figure S8. HDX-MS volcano plots of USP30 in complex with USP30-I-1 and USP30$_{inh}$. A greater overall solvent protection is observed for USP30 in the presence of the non-covalent inhibitor, as compared to its covalent counterpart. **USP30-I-1** primarily induces solvent protection in the region encompassing the catalytic Cys77.
